# Caffeine makes a splash: a systematic review and multilevel meta-analysis exploring the effects of caffeine intake on swimming performance

**DOI:** 10.1080/15502783.2026.2692016

**Published:** 2026-06-21

**Authors:** Ziyu Wang, Bopeng Qiu, Jinxuan Bao, Penglin Diao, Michael Hamlin, Juan Del Coso, Jozo Grgic, Carl Petersen

**Affiliations:** a Faculty of Health, University of Canterbury, Christchurch, New Zealand; b Division of Sports Science and Physical Education, Tsinghua University, Beijing, People's Republic of China; c Sports Coaching College, Beijing Sport University, Beijing, People's Republic of China; d School of Sports Training, Wuhan Sports University, Wuhan, People's Republic of China; e Department of Tourism, Sport, and Society, Lincoln University, Lincoln, New Zealand; f Sport Sciences Research Centre, Rey Juan Carlos University, Fuenlabrada, Spain; g Institute of Health and Sport Sciences, Faculty of Health Sciences, Universidad Francisco de Vitoria, Madrid, Spain; h Department of Sports Science and Physical Education, The Chinese University of Hong Kong, Hong Kong, People's Republic of China

**Keywords:** Caffeine, swimming performance, three-level meta-analysis, blood lactate

## Abstract

**Background:**

Evidence for acute caffeine supplementation in swimming remains inconsistent across reviews, likely due to between-study heterogeneity and methodological differences.

**Methods:**

Five databases were searched for randomized placebo-controlled crossover trials exploring the effects of caffeine on swimming performance in competitive swimmers. Performance outcomes were pooled as standardized mean differences (SMD; Hedges’ g), with time outcomes sign-reversed so that positive values indicated improved performance. Dependence among multiple outcomes within studies was addressed using three-level random-effects models (REML). As a supplementary, interpretable metric, log ratios of means (lnRoM) were meta-analysed and expressed as percentage change. Post-exercise blood lactate (mmol/L) was pooled as mean differences (MD). Risk of bias was assessed using RoB 2 and certainty of evidence using GRADE. Prespecified exploratory moderator analyses examined potential effect modification (dose, timing, distance, administration form, stroke, gender, and athlete level).

**Results:**

Thirteen studies were included (144 men and 48 women), which contributed 28 performance effect sizes. Caffeine improved swimming performance (SMD = 0.57, 95% CI: 0.20 to 0.94; *p* = 0.005), corresponding to a +1.71% improvement in lnRoM (95% CI: +1.01% to +2.41%; *p* < 0.001). Heterogeneity was substantial (I²_total_ = 64.7%) and primarily between studies. Eight studies contributed 24 blood lactate effect sizes. In the meta-analysis, caffeine increased post-test lactate (MD = 0.85 mmol/L, 95% CI: 0.22 to 1.49; *p* = 0.016) with high heterogeneity (I²_total_ = 76.5%). Exploratory moderator analyses suggested a possible dose-related pattern for swimming performance, with larger pooled effects at ≥6 mg/kg (SMD = 0.95, 95% CI: 0.52 to 1.38) than at <6 mg/kg (SMD = 0.22, 95% CI: −0.15 to 0.60; *p*_difference_ = 0.017). Other performance moderators did not show clear between-subgroup differences. For lactate, exploratory subgroup analyses suggested larger increases in freestyle and short-distance events, although these patterns were more sensitive to modelling assumptions and should be interpreted cautiously.

**Conclusion:**

Acute caffeine ingestion confers a moderate ergogenic benefit for swimming performance (SMD = 0.57, 1.7% improvement) and is associated with higher post-test blood lactate (MD = 0.85 mmol/L). Dose (≥6 mg/kg) may be associated with larger performance benefits, whereas lactate responses appear more pronounced in freestyle and shorter events. However, these subgroup findings are hypothesis-generating and require exploration.

## Graphical abstract

**Figure:**
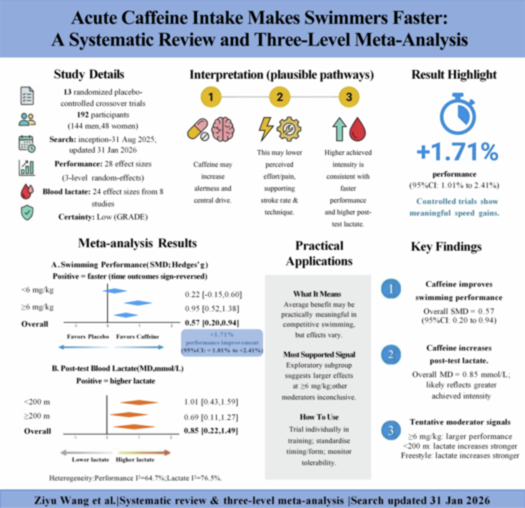

## 1. Introduction

The main objective in competitive swimming is to complete the race as quickly as possible [1]. Swimming races are frequently tightly contested, and changes in performance time as small as 0.5-0.6% can affect placing [2,3]; therefore, even small ergogenic effects may be practically meaningful in competition [4]. In this context, swimmers commonly use sports supplements as one of several strategies to optimise performance, particularly in high-level competition [5]. Competitive swimmers commonly report consuming caffeine as a performance-related supplement, with approximately half of athletes in one study indicating regular use of caffeine products [5]. Consistent with these findings, in-competition urine monitoring data show that median urinary caffeine concentration in aquatics increased from 0.1 µg/mL in 2004 to 0.7 µg/mL in 2015 [6], suggesting an increase in caffeine use. Available evidence supports caffeine’s ergogenic effects in various sports disciplines and competitive scenarios [7,8]. From a physiological perspective, caffeine may exert its effects through multiple pathways, including enhancing central nervous system arousal and motor drive, promoting catecholamine-related responses, and improving skeletal muscle excitation-contraction coupling and contractile capacity [9,10]. In addition to performance outcomes, acute caffeine intake may also influence post-exercise physiological responses such as increasing blood lactate concentration [11].

Several meta-analyses have explored the effects of caffeine on swimming performance but provided inconsistent conclusions. Importantly, although the direction of effect across previous reviews was generally similar (favouring caffeine), several of the previous meta-analyses actually did not find significant differences between the conditions. Specifically, one meta-analysis examined the effects of caffeine on 25-m and 50-m swimming times [12]. Although no statistically significant differences were observed between caffeine and placebo conditions, the corresponding 95% confidence intervals (CI) were very wide. This imprecision reflects the very limited evidence base, as the analyses were based on only two studies for the 25-m distance and three studies for the 50-m distance. Other syntheses [13] of evidence exploring caffeine’s effects on swimming performance used a network meta-analysis and focused more broadly on supplements that may improve swimming performance. This analysis also did not find significant improvement in 100 and 200-m swimming performance from caffeine intake. However, only five studies involving caffeine were included, potentially reducing the robustness of caffeine-related estimates and increasing uncertainty in comparative rankings. Another review [14] restricted inclusion to higher-level swimmers, which substantially reduced the available evidence base. As a result, only two eligible trials were included, resulting in low statistical power.

While previous reviews were generally directionally consistent in suggesting a potential ergogenic effect of caffeine, statistically significant effects on swimming performance were reported in only one meta-analysis, with the remaining reviews limited by sparse outcome-specific data and differences in analytical scope. In contrast, only one meta-analysis of eight studies reported an overall ergogenic effect of caffeine on swimming performance, but did not explore the influence of potential moderators such as training status, caffeine dose, mode of administration, or timing of consumption [15]. Additionally, this analysis included only studies reporting swimming time but not swimming velocity and also included one study that focused on triathlon performance, not swimming in isolation. To help contextualise the present review, the main characteristics of previous meta-analyses on caffeine and swimming performance are summarised in Supplementary Table S1.

Taken together, existing syntheses are limited by small and selective evidence bases, wide statistical uncertainty, and restricted analytical scope. Additionally, previous meta-analyses have not resolved whether the efficacy of caffeine supplementation is mediated by factors such as dose, timing of ingestion, swimming level, or other study characteristics. In particular, previous reviews have provided limited or no detailed subgroup and meta-regression results, leaving unresolved whether caffeine’s ergogenic effect varies according to dose, timing, swimmer level, stroke, or race distance. These limitations indicate the need for a comprehensive and methodologically robust synthesis.

Therefore, the aim of this review was to conduct a systematic review coupled with a multilevel meta-analysis to examine the acute effects of caffeine on swimming performance. Our secondary aims were to: (i) synthesise the effects of caffeine on post-exercise blood lactate concentration, and (ii) explore potential moderators of caffeine’s performance effects, such as training status, swimming distance, and caffeine consumption protocol. We hypothesised that acute caffeine supplementation would improve swimming performance, with magnitudes varying according to event characteristics, caffeine dosing strategies, and athlete- or study-level factors. The rationale for the present review lies not only in updating the evidence base, but also in providing a broader and more statistically robust synthesis of caffeine’s effects in swimming while exploring potential sources of between-study variability.

## 2. Method

### 2.1. Search strategy

This systematic review was conducted in accordance with the Preferred Reporting Items for Systematic Reviews and Meta-Analyses (PRISMA) guidelines and was registered in PROSPERO (CRD420261278143) [16]. A comprehensive search strategy was developed using a combination of Medical Subject Headings (MeSH) and free-text terms covering key concepts related to caffeine and swimming performance. No database filters or restrictions were applied to the search. We searched PubMed/MEDLINE, Embase, Cochrane Library, Web of Science, and SPORTDiscus from database inception to 31 August 2025, and updated the search on 31 January 2026. The search syntax applied in these databases was: (caffeine OR coffee OR “energy drink*” OR caffeinat* OR “caffeinated gum”) AND (swim* OR swimmer* OR “swimming performance”). All records were exported to EndNote 20 (Clarivate Analytics, London, UK) for further review. As part of secondary searches, we screened the reference lists of included studies and other relevant reviews [12,15]. The literature search and study screening were performed independently by two reviewers (Z.W. and J.B.), with any disagreements resolved through discussion and consultation with a third reviewer (B.Q.). Agreement between the two reviewers during study selection was quantified using Cohen’s kappa at the title/abstract screening stage and the full-text eligibility stage.

### 2.2. Inclusion criteria

Articles were deemed eligible if they met the following predetermined PICOS criteria:

Participants (*P*): competitive swimmers of any age or gender.

Intervention (I): acute caffeine administration delivered via oral ingestion (capsules/tablets/drinks) or mouth rinse.

Comparison (C): a placebo condition matched as closely as possible to the caffeine condition but without active caffeine (or a decaffeinated control, where applicable).

Outcomes (O): Primary outcomes were swimming performance measures, including time-trial/race completion time, swimming velocity, and standardised swimming performance tests (including repeated-sprint/intermittent protocols). Secondary outcomes included post-exercise blood lactate concentration (mmol/L).

Study design (S): single-or-double blind randomised and placebo-controlled crossover trials that were published in English or other languages, provided that sufficient data were available for extraction.

### 2.3. Data extraction

The following data were extracted from the included studies: (a) lead author name, year of publication, and study design; (b) participant characteristics (sample size, age, gender, and competitive/training level); (c) caffeine administration (dose and form of intake, where available); (d) timing of ingestion relative to the start of the swimming performance test; and (e) swimming performance testing context (test type, swimming distance, and key features of the testing procedure, such as pool setting and number of trials/repetitions, where reported). For each placebo–caffeine comparison within the included studies, we extracted the mean, standard deviation, and sample size independently for both the caffeine condition and the placebo/control condition. When multiple outcomes or conditions were reported within a study (e.g. different distances or doses), each eligible comparison was extracted as a separate effect size with a unique identifier. Two reviewers (Z.W. and J.B.) independently performed data extraction. When outcome data were presented only in figures, plots were digitised using WebPlotDigitizer. Discrepancies in extracted data were resolved through discussion, with involvement of a third reviewer (B.Q.) when necessary.

### 2.4. Risk of bias and certainty of evidence

Risk of bias was assessed using the Cochrane Risk of Bias 2 tool adapted for crossover trials [17]. In addition to the standard RoB 2 domains, we evaluated bias arising from period and carryover effects, including sequence balance, handling of period effects where relevant, adequacy of washout, and the possibility of selective reporting of first-period results.

The certainty of evidence was assessed using the Grading of Recommendations Assessment, Development, and Evaluation (GRADE) system [18], which classifies evidence as high, moderate, low, or very low.

Two reviewers (Z.W. and B.Q.) independently conducted the assessments, and any disagreements were resolved through discussion or, when necessary, consultation with a third reviewer (J.B.).

### 2.5. Data analysis

All swimming performance outcomes (e.g. race/time-trial completion time or swimming velocity) were directionally harmonised so that positive effects consistently indicated improved performance. The primary synthesis used standardised mean differences (SMDs). The magnitude of SMDs was interpreted using Cohen’s conventional thresholds, with values <0.2 considered trivial, 0.2 to <0.5 small, 0.5 to <0.8 moderate, and ≥0.8 large [19]. To aid practical interpretation, we additionally computed ratios of means (RoM; analysed on the log scale) and expressed these as percentage changes for reporting [20]. For the post-exercise blood lactate concentration, we pooled effects as mean differences (MD) using the original units (mmol/L); these effects were interpreted according to the original direction of measurement.

To account for statistical dependence arising from multiple effect sizes extracted from the same study, effect sizes were synthesised using a three-level random-effects meta-analysis [21]. Effect sizes were nested within studies, with sampling variance at level 1, within-study heterogeneity at level 2, and between-study heterogeneity at level 3. Models were fitted using restricted maximum likelihood (REML) estimation. For pooled effects, we reported the overall estimated effect size and corresponding 95% CI. When multiple outcomes or conditions were reported within a study (e.g. different distances, doses, repeated trials, or repetitions within an intermittent set), each eligible comparison was extracted as a separate effect size with a unique identifier.

For paired/crossover comparisons, when the standard deviation of the within-participant differences (SD_diff_) was not reported, we followed Cochrane guidance [17] for crossover trials by deriving or imputing the within-subject correlation coefficient (*r*) to obtain SD_diff_. When paired t-test statistics were available, SD_diff_ was derived from the paired t-test identity:

(1) $$t=\frac{MD}{{SD}_{d\mathrm{iff}}/\sqrt{n}}\;$$

and hence:

(2) $$\mathrm{SDdiff}=\frac{\left|\mathrm{MD}\right|\sqrt{n}}{\left|t\right|}$$

The corresponding within-subject correlation was then calculated as:

(3) $$r=\;\frac{SD_{\mathrm{placebo}}^{2}+SD_{\mathrm{caffeine}}^{2}{-SD}_{\mathrm{diff}}^{2}}{2SD_{\mathrm{placebo}}SD_{\mathrm{caffeine}}}$$

When the within-subject correlation could not be derived directly, we imputed it using values estimated from studies with sufficient paired information. For blood lactate, one study [22] allowed direct estimation (*r* = 0.89). For swimming performance, three outcomes from two studies [23,24] yielded *r* values of 0.92, 0.93 and 0.99; we used the lowest of these empirically derived values (*r* = 0.92) for the primary analysis. To assess robustness to the assumed within-subject correlation, we repeated the analyses using alternative plausible values (*r* = 0.50, 0.70, and 0.90). These sensitivity analyses were used not only to examine the stability of the pooled effects, but also to evaluate whether the general pattern of exploratory subgroup and meta-regression findings was materially altered under alternative assumptions.

We also conducted sensitivity analyses to ensure that the pooled effect was not driven by the inclusion of multiple outcomes per study. To be specific, we conducted a study-level aggregation sensitivity analysis by combining all effect sizes within each study into a single inverse-variance–weighted estimate and re-ran a random-effects model [25]. In addition, we performed leave-one-out analyses, iteratively excluding each effect size to evaluate the influence of individual data points on the pooled estimate [26]. Finally, we conducted an additional sensitivity analysis excluding studies that used caffeine mouth rinse, coffee, or energy drinks, because these interventions may complicate interpretation of whether the observed effects are attributable to conventional systemic caffeine exposure.

To explore potential sources of heterogeneity and examine effect modification, we conducted subgroup analyses and mixed-effects meta-regression models using REML. Because several subgroup categories were sparse and some protocol characteristics were correlated at the study level, subgroup and meta-regression analyses were considered exploratory a priori. No formal multiplicity correction was applied to these analyses; accordingly, findings were interpreted cautiously as hypothesis-generating rather than confirmatory. Subgroup analyses were performed for categorical moderators, and between-subgroup differences were formally tested using the omnibus moderator test (QM). For continuous moderators with sufficient variation, we performed meta-regression analyses focusing on caffeine dose (mg/kg), time between ingestion and performance testing (min), and swimming distance (m). To assess the functional form of the association, we fitted both linear and flexible non-linear models (e.g. quadratic terms or restricted cubic splines) and compared model fit using Akaike Information Criterion (AIC). Meta-regression was considered only when more than 10 studies were available to reduce the risk of spurious associations [17]. Meta-regression results were presented as predicted effects across the observed range of the moderator with 95% CI.

Subgroup analyses were also conducted to explore potential effect modifiers, including stroke (freestyle vs. preferred stroke), caffeine dose (mg/kg), timing of ingestion (minutes before testing), administration form (capsules/tablets, drinks, mouth rinse where available), athlete level (highly trained/national-level vs. trained/developmental athletes), gender (male vs female), and distance (short vs middle-to-long distance). For blood lactate only, subgroup analyses were conducted to explore potential effect modifiers, including blood lactate sampling time point (time bin; minutes post-test), stroke (freestyle vs preferred stroke), caffeine dose (mg/kg), timing of ingestion (minutes before testing), administration form (capsules/tablets, drinks, mouth rinse where available), athlete level (highly trained/national-level vs trained/developmental athletes), gender (male vs female), and distance (short vs middle-to-long distance). These subgroup classifications were defined a priori based on physiological plausibility and common practice/recommendations in the caffeine literature and were constrained by the reporting granularity of the included studies. When caffeine dose was reported as an absolute amount (mg), we converted it to a body-mass–normalised dose (mg/kg) using the mean body mass reported for the sample. Detailed coding rules and supporting references for each classification are provided in Supplementary file 1.

Small-study effects and potential publication bias were examined using funnel plots and regression-based asymmetry tests. Funnel plots were visually inspected by plotting effect sizes against their standard errors. We evaluated heterogeneity of the analyses using Q and I^2^ statistics [27]. Analyses were performed using the metafor package in R, version 4.4.0. The statistical significance threshold was set at *p* < 0.05.

## 3. Results

### 3.1. Search outcomes

**Figure 1.**
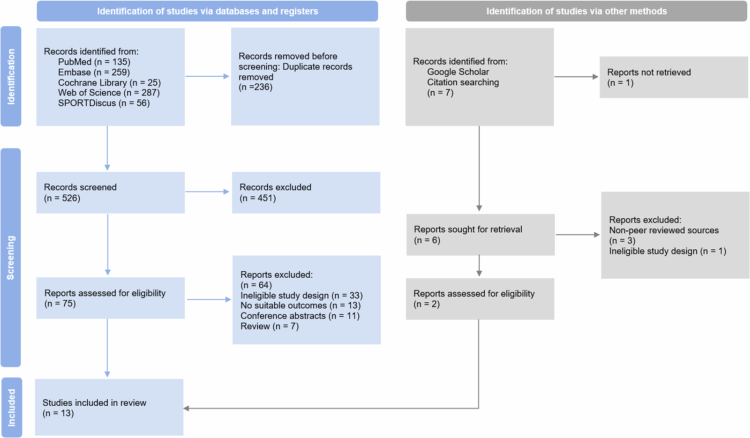
PRISMA 2020 flow diagram showing the identification, screening, eligibility assessment, and inclusion of studies.

We identified 762 records through database searching (PubMed/MEDLINE *n* = 135; Embase *n* = 259; Cochrane Library *n* = 25; Web of Science *n* = 287; SPORTDiscus *n* = 56). After removing 236 duplicates, 526 records were screened by title and abstract, and 451 were excluded. Seventy-five full-text reports were assessed for eligibility and 64 were excluded for the following reasons: ineligible study design (*n* = 33), no suitable outcomes (*n* = 13), conference abstracts (*n* = 11), and reviews (*n* = 7). In addition, 7 records were identified through Google Scholar/citation searching. Of these, 1 record was available only as an abstract and no full text could be obtained. The remaining 6 reports were assessed, of which 4 were excluded (non-peer-reviewed sources, *n* = 3; ineligible study design, *n* = 1), and 2 studies were included in the review. Finally, 13 studies [22–24,28–37] were included in the review (Figure 1). Inter-reviewer agreement was substantial for title/abstract screening (k = 0.88) and for full-text eligibility assessment (k = 0.95).

### 3.2. Study characteristics

In the 13 studies included in this systematic review, there was a total sample of 192 participants, including 144 men and 48 women. Of these, six studies included only men, two included only women, and five recruited mixed-gender samples. Based on training status, 28 participants (Tier 3) were classified as national-level athletes, whereas 164 participants (Tier 2) were developmental athletes. All studies used a randomised placebo-controlled design. Regarding caffeine administration, eight studies reported body-mass–normalised doses (range: 3 to 6.2 mg/kg). Caffeine was delivered predominantly as capsules/tablets (*n* = 9) or drinks (*n* = 3), and one study used a mouth rinse protocol. The remaining three studies administered absolute caffeine doses (range: 250 to 750 mg) using a drink, capsules, or mouth rinse. Caffeine was typically provided 30 to 150 min before the swimming trials (most commonly 45 to 60 min), while one study used a mouth rinse protocol immediately prior to the start of the trial. Outcomes included swimming performance assessed as time (25 m, 50 m, 100 m, 200 m, 400 m, 1500 m, and 50 yd) and swimming velocity (e.g. 100 m velocity and 10 × 400 m swim velocity). The general characteristics of the included studies are presented in Table 1.

**Table 1.** Study and participant characteristics and caffeine intervention details of included trials examining the acute effects of caffeine on swimming performance in trained athletes.

Study	Sample size (M/F)	Age (years)	Caffeine dosage	Caffeine form	Timing of intake (before exercise)	Placebo	Outcomes	Stroke
Alkatan 2020 Kuwait [28]	18 (18/0)	18–25	250 mg	Drink	45 min	Decaf coffee	25 m time	Freestyle
Bostan 2023 Türkiye [29]	48(48/0)	20 ± 2	750 mg	Mouth rinse	Immediately	Carbohydrate	25 m time 50 m time	Freestyle
Collomp 1992 France [30]	7 (3/4)	17 ± 2.1	250 mg (≈ 4.3 mg/kg)	Capsules	60 min	Mineral inert	100 m velocity Blood lactate	Freestyle
Goods 2017 Australia [22]	9 (9/0)	20.8 ± 2.8	3 mg/kg	Tablets	60 min	Non-nutritive sweetener	6 × 75 m time	Freestyle
Hosseininejad 2017 Iran [36]	14 (0/14)	24.6 ± 4	6 mg/kg	Capsules	60 min	Carbohydrate	50 m time 400 m time Blood lactate	Freestyle
Kürşat 2024 Turkey [23]	8 (0/8)	21.3 ± 1.4	6 mg/kg	Capsules	60 min	Fibre-based	25 m time 50 m time	Freestyle
Lara 2015 Spain [31]	14 (14/0)	20.2 ± 2.6	3 mg/kg	Drink	60 min	Placebo energy drink	50 m time Blood lactate	Preferred stroke
Longo 2010 Brazil [37]	9 (6/3)	16.00 ± 1.32	5 mg/kg	Capsules	60 min	Carbohydrate	100 m time Blood lactate	Freestyle
MacIntosh 1995 Canada [32]	11 (7/4)	21.8 ± 0.5	6 mg/kg	Drink	150 min	Non-nutritive sweetener	1500 m time Blood lactate	Freestyle
Newbury 2022 United Kingdom [33]	8 (5/3)	16–19	3 mg/kg	Capsules	60 min	Carbohydrate	100 m time Blood lactate	Preferred stroke
Pruscino 2008 Australia [35]	6 (6/0)	NR	6.2 ± 0.3 mg/kg	Tablets	45 min	Carbohydrate	200 m time Blood lactate	Freestyle
Salgueiro 2022 Brazil [34]	10 (10/0)	18.2 ± 1.7	6 mg/kg	Capsules	60 min	6 mg/kg/carbohydrates	10 × 400 m velocity Blood lactate	Freestyle
Vanata 2014 USA [24]	30 (18/12)	19.5 ± 1.4	3 mg/kg	Capsules	30 min	NR	50-yard time	Freestyle

Note: BM = body mass; M/F = male/female; NR = not reported.

### 3.3. Methodological quality of included studies

**Figure 2.**
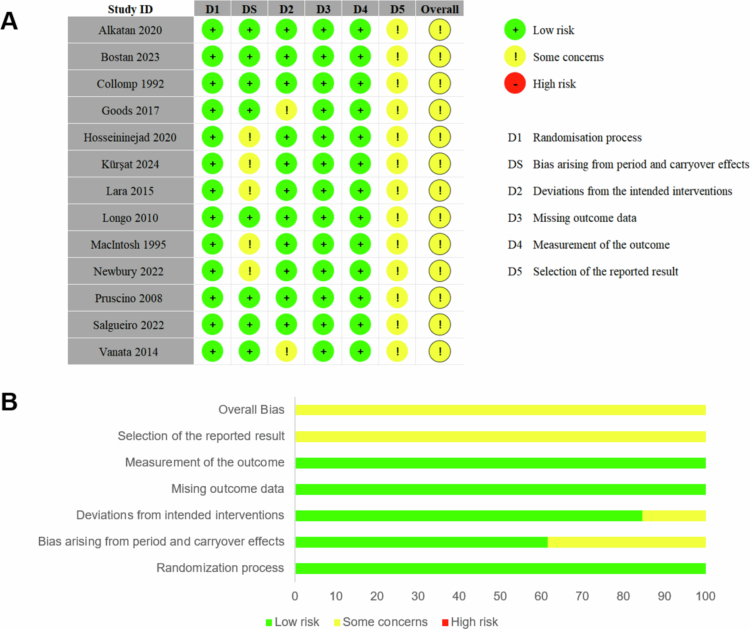
Risk-of-bias assessment of the included studies using the Cochrane RoB 2 tool for crossover trials. (A) Domain-level and overall risk-of-bias judgements for each included study. (B) Summary of the proportions of studies judged as low risk, some concerns, or high risk for each domain and overall. D1, bias arising from the randomisation process; DS, bias arising from period and carryover effects; D2, bias due to deviations from intended interventions; D3, bias due to missing outcome data; D4, bias in measurement of the outcome; D5, bias in selection of the reported result.

As shown in Figure 2, all studies were judged as low risk for bias arising from the randomisation process (D1), missing outcome data (D3), and measurement of the outcome (D4). For bias arising from period and carryover effects (DS), 8 studies were judged as low risk and 5 studies as having some concerns [23,31–33,36]. For bias due to deviations from intended interventions (D2), 11 studies were judged as low risk and 2 studies as having some concerns [22,24]. For bias in selection of the reported result (D5), all studies were judged as having some concerns. Consequently, the overall risk-of-bias judgement for all included studies was some concerns.

### 3.4. Meta-analysis results

#### 3.4.1. Swimming performance

**Figure 3.**
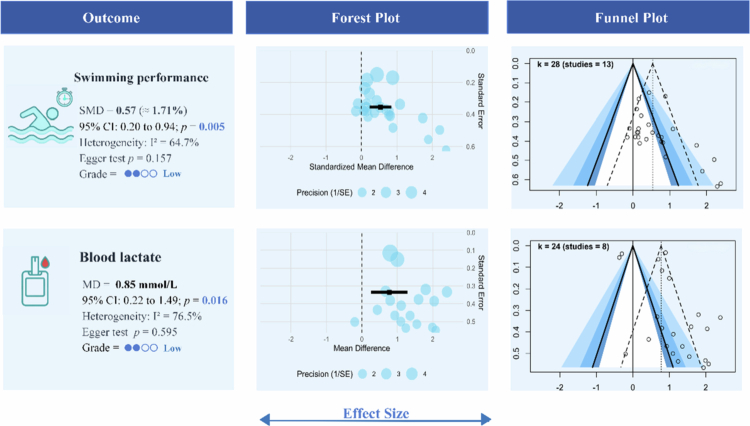
Effects of caffeine on swimming performance and blood lactate. The middle panels show forest-style bubble plots (orchard plots) of individual effect sizes (bubble size proportional to inverse-variance weight) with the pooled estimate and its 95% confidence interval. The right panels show contour-enhanced funnel plots based on effect size level aggregated effects.

We pooled 28 effect sizes from 13 studies in the primary three-level meta-analysis, which showed a moderate ergogenic effect favouring caffeine (SMD = 0.57, 95% CI: 0.20 to 0.94; *p* = 0.005; Figure 3). There was evidence of heterogeneity (Q(27) = 93.98, *p* < 0.001). In the three-level model, heterogeneity was substantial (I²_total_ = 64.7%), with most variability attributable to between-study differences (I²_level3_ = 51.2%) rather than within-study, between-effect differences (I²_level2_ = 13.4%). The estimated variance components were τ²_level3_ = 0.254 (*τ* = 0.504) and τ²_level2_ = 0.066 (*τ* = 0.258). Visual inspection of the contour-enhanced funnel plot did not indicate asymmetry, and Egger’s regression based on study-level aggregated effects showed no evidence of small-study effects (z = 0.86; *p* = 0.157; Figure 3). Figure 3 summarises the GRADE certainty ratings for swimming performance. The certainty of evidence was judged low. Detailed domain-level decisions and explanations are available in Supplementary Table S2.

For interpretability, the supplementary ratio-of-means analysis yielded lnRoM = 0.017 (95% CI: 0.010 to 0.024; *p* < 0.001), equivalent to a + 1.71% performance improvement (95% CI: + 1.01% to + 2.41%; Supplementary Figure S1). Effects were direction-aligned such that positive values indicated improved performance with caffeine.

#### 3.4.2. Blood lactate after swimming test

We pooled 24 effect sizes from 8 studies in a three-level meta-analysis, showing higher post-exercise blood lactate concentration following caffeine compared with placebo (MD = 0.85 mmol/L, 95% CI: 0.22 to 1.49; *p* = 0.016; Figure 3). There was evidence of substantial heterogeneity (QE(23) = 970.34, *p* < 0.001). In the three-level model, heterogeneity was high (I²_total_ = 76.5%), with most variability attributable to between-study differences (I²_level3_ = 52.7%) and the remainder to within-study, between-effect differences (I²_level2_ = 23.8%). The estimated variance components were τ²_level3_ = 0.395 (*τ* = 0.628) and τ²_level2_ = 0.179 (*τ* = 0.423). Visual inspection of the contour-enhanced funnel plot did not suggest asymmetry, and Egger’s regression test based on study-level aggregated effects showed no evidence of small-study effects (z = 0.53; *p* = 0.595; Figure 3). Figure 3 summarises the GRADE certainty ratings for blood lactate, and certainty of evidence was judged low. Detailed domain-level decisions and explanations are available in Supplementary Table S2.

#### 3.4.3. Moderator analyses

##### 3.4.3.1. Subgroup analyses.

**Figure 4.**
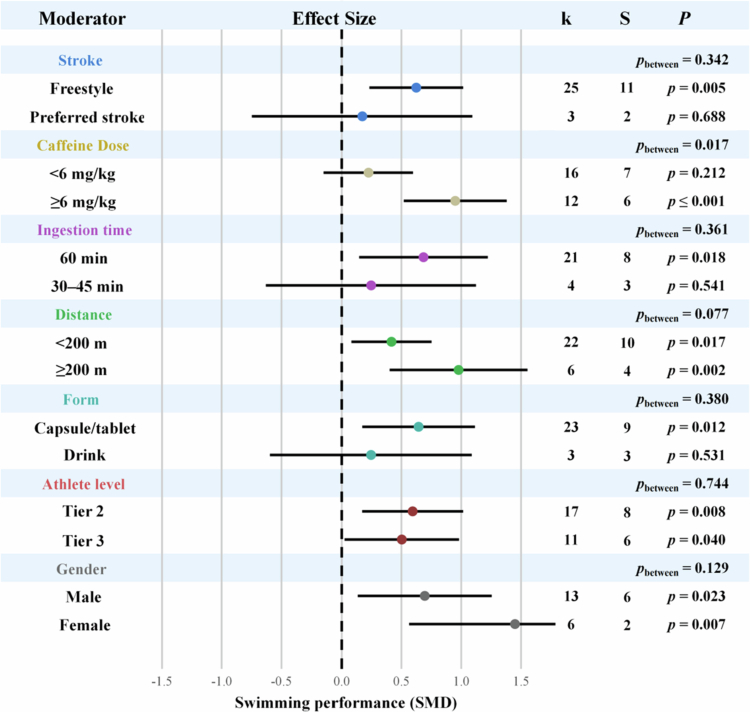
Subgroup analyses exploring caffeine’s effects on swimming performance from mixed-effects multilevel models. Coloured points indicate pooled subgroup standardised mean differences (SMDs) and horizontal bars show 95% confidence intervals (CIs). The dashed vertical line marks no effect (SMD = 0). k = number of effect sizes; S = number of studies. *P* (within) tests the pooled subgroup effect versus 0. *P* (between) reports the omnibus moderator test for between-subgroup differences.

We explored potential effect modification using mixed-effects multilevel meta regression models (Figure 4).

Caffeine dose demonstrated evidence of effect modification. The moderator test indicated a significant difference between dose categories (F(1,11) = 7.87, *p* = 0.017). The pooled effect was larger in the ≥6 mg/kg subgroup (SMD = 0.95, 95% CI: 0.52 to 1.38) than in the <6 mg/kg subgroup (SMD = 0.22, 95% CI: −0.15 to 0.60), with an estimated between-group contrast of 0.73 SMD units (95% CI: 0.16 to 1.29). Besides, the moderator test indicated a marginal difference between distance categories (F(1,26) = 3.39, *p* = 0.077). The pooled effect tended to be larger in the middle-to-long (≥200 m) subgroup (SMD = 0.98, 95% CI: 0.40 to 1.55) than in the short distance (<200 m) subgroup (SMD = 0.42, 95% CI: 0.08 to 0.75), with an estimated between-group contrast of 0.56 SMD units (95% CI: −0.07 to 1.19).

Other indicators, including athlete level, gender, stroke category, ingestion timing, and administration form, also did not show evidence of between-subgroup differences (all *p* > 0.05). Although statistically significant pooled effects were observed in some individual subgroups (e.g. freestyle, 60 min, capsule/tablet), these findings were not interpreted as evidence of subgroup differences in the absence of a significant formal between-subgroup test.

**Figure 5.**
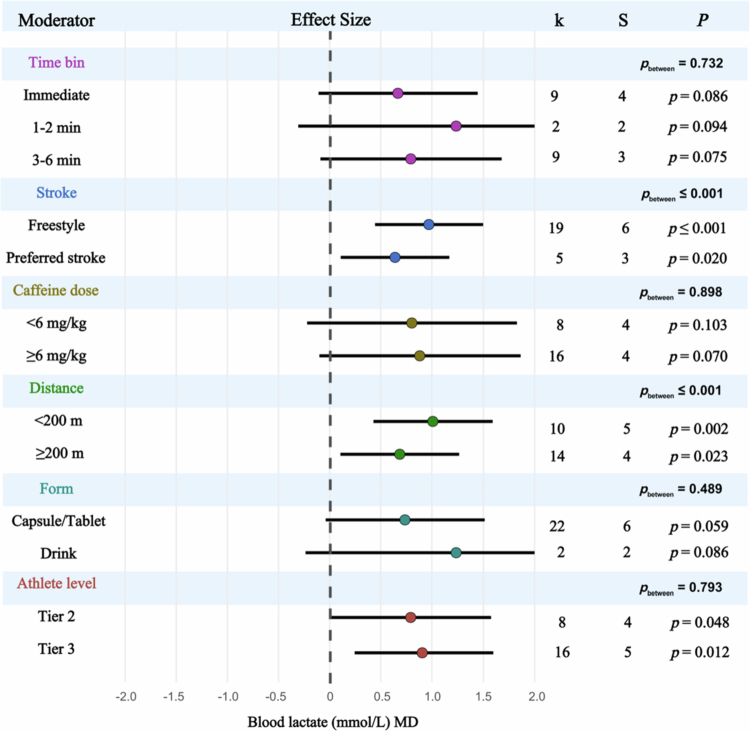
Subgroup analyses exploring caffeine’s effects on blood lactate from mixed-effects multilevel models. Coloured points indicate pooled subgroup mean differences (MDs) and horizontal bars show 95% confidence intervals (CIs). The dashed vertical line marks no effect (MD = 0). k = number of effect sizes; S = number of studies. *P* (within) tests the pooled subgroup effect versus 0. *P* (between) reports the omnibus moderator test for between-subgroup differences.

For blood lactate outcomes, we also explored potential effect modification using mixed-effects multilevel meta-regression models (Figure 5).

Exploratory subgroup analyses suggested between-subgroup differences for stroke category and distance. The pooled effect was larger in freestyle (MD = 0.97, 95% CI: 0.44 to 1.50) than in preferred stroke (MD = 0.64, 95% CI: 0.11 to 1.17), and larger in short-distance events (MD = 1.01, 95% CI: 0.43 to 1.59) than in middle-to-long-distance events (MD = 0.69, 95% CI: 0.11 to 1.27). However, these findings should be interpreted cautiously because the analyses were exploratory, several subgroup categories were sparse, and statistical support for these patterns was attenuated under the more conservative assumed within-subject correlation in sensitivity analyses.

No evidence of effect modification was observed for post-exercise blood lactate sampling time window (time bin), caffeine dose category, administration form, or athlete level (*p* > 0.05). Although some within-subgroup pooled effects were statistically significant (e.g. 30–45 min: MD = 1.58, 95% CI: 0.12 to 3.05; Tier 2: MD = 0.79, 95% CI: 0.01 to 1.57; Tier 3: MD = 0.90, 95% CI: 0.22 to 1.57), the corresponding between-subgroup tests were non-significant, and some comparison subgroups were sparse, indicating limited precision rather than clear effect modification.

Planned subgroup contrasts for ingestion timing and gender were not formally tested because, after applying minimum data requirements (only 1 study per subgroup level), insufficient information remained to support a valid between-subgroup comparison. Although some within-subgroup pooled effects were statistically significant, the corresponding between-subgroup tests were non-significant, and these findings were therefore not interpreted as clear evidence of effect modification.

##### 3.4.3.2. Meta regression.

**Figure 6.**
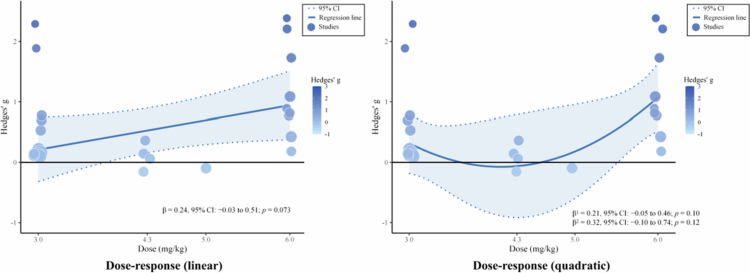
Dose–response meta-regression of caffeine dose (mg/kg) on swimming performance (Hedges’ g). Circles represent individual effect sizes; circle size reflects inverse-variance weight (precision), and colour indicates the observed Hedges’ g. The solid line shows the fitted meta-regression, and the shaded area (bounded by dotted lines) denotes the 95% confidence interval. The horizontal line marks no effect (g = 0). Left panel: linear specification; right panel: quadratic specification. Model coefficients (*β*) with 95% CIs and *p*-values are shown within panels. We calculated and compared the Akaike Information Criterion (AIC) for both models; the right panel (quadratic model) yielded a slightly lower AIC (ΔAIC < 2), indicating similar model support and only weak evidence for a better fit.

**Figure 7.**
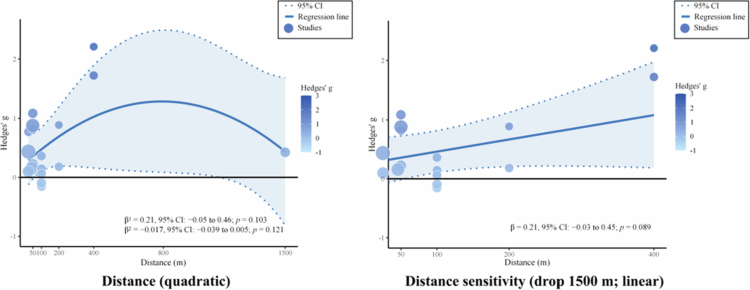
Meta-regression of swimming distance (m) on the effect of caffeine on swimming performance (Hedges’ g). Circles represent individual effect sizes; circle size reflects inverse-variance weight (precision) and colour indicates the observed Hedges’ g. The solid line shows the fitted meta-regression; the shaded band represents the 95% confidence interval, with dotted lines indicating its bounds. The horizontal line marks no effect (g = 0). Left panel shows the quadratic specification fitted to all distances (including 1500 m), whereas the right panel shows a sensitivity analysis excluding the single 1500-m effect size, for which the linear specification was best supported by model fit. Model coefficients (*β*) with 95% CIs and *p*-values are shown within panels.

Alternative non-linear specifications (quadratic and restricted cubic spline models) were examined as exploratory analyses. Model fit was compared using ML-derived AIC and likelihood ratio tests against the linear specification. Across models, AIC differences were small (all ΔAIC < 2) and the likelihood ratio tests provided at most borderline evidence for improved fit (all *p* ≥ 0.059, Figure 6, Figure 7, Supplementary Figure S2). Therefore, inference focused on the overall direction and magnitude of the association, and any apparent non-linear patterns were interpreted cautiously.

Dose (mg/kg; k = 26 effects from 12 studies; four distinct dose levels) was examined as a mean-centred continuous moderator. Competing functional forms (linear, quadratic, and a 2-df natural spline) were compared using ML-based AIC and likelihood ratio tests against the linear specification. The quadratic model yielded the lowest AIC (AIC = 63.23), but the improvement over the linear model was small (ΔAIC = 1.57) and not clearly supported by the LRT (*p* = 0.059). The natural spline performed similarly (AIC = 63.35; ΔAIC = 0.12; LRT vs linear *p* = 0.063). In an exploratory quadratic multilevel meta-regression (including β^1^ and β²), both terms were imprecisely estimated (β^1^ = 0.21, 95% CI: −0.05 to 0.46; *p* = 0.10; β^2^ = 0.32, 95% CI: −0.10 to 0.74; *p* = 0.12), and the joint moderator test was not statistically significant (*p* = 0.064). For interpretability, we report the slope from a separate linear model: *β* = 0.24 SMD per 1 mg/kg (95% CI: −0.03 to 0.51; *p* = 0.073), suggesting a possible positive dose–response trend, although uncertainty remained (Figure 6).

Timing (time before testing) (k = 26 effects from 12 studies; four distinct timing values) was examined as a mean-centred continuous moderator (Supplementary Figure S2). A linear specification was retained because it provided the best ML-based AIC (AIC = 68.97), and neither a quadratic nor a 2-df natural spline improved fit versus the linear model (ΔAIC ≈ 0.84–0.85; *p* ≈ 0.28). In the REML multilevel meta-regression, the association with timing was negligible and not statistically significant (*β* = 0.03 per unit increase in timing, 95% CI: −0.46 to 0.52; *p* = 0.89), indicating no evidence of effect modification by ingestion timing. Results were unchanged after excluding the 150-min condition (*β* = 0.63, 95% CI: −0.85 to 2.10; *p* = 0.36).

Distance (per 100 m; k = 21 effects from 11 studies; seven distinct distances) was examined as a mean-centred continuous moderator (Figure 7). Competing functional forms (linear, quadratic, and a 2-df natural spline) were compared using ML-based AIC and likelihood ratio tests against the linear specification. The quadratic model yielded the lowest AIC (AIC = 46.55), but differences in model fit were small (ΔAIC ≤ 0.86) and the LRT provided no clear support for either the quadratic or spline specification versus linear (*p* = 0.091 and *p* = 0.101, respectively). In the REML quadratic multilevel meta-regression, the joint moderator test was non-significant (*p* = 0.254) and both terms were imprecisely estimated (β^1^ = 0.21, 95% CI: −0.05 to 0.46; *p* = 0.103; β^2^ = −0.017, 95% CI: −0.039 to 0.005; *p* = 0.121). For interpretability, we also report the linear slope, which was close to zero and non-significant (*β* = 0.02 SMD per 100 m, 95% CI: −0.07 to 0.11; *p* = 0.617), indicating no clear evidence that race distance modified the effect. In a sensitivity analysis excluding the single 1500-m datapoint, the linear model provided the best fit (AIC = 43.86) and the slope became larger but remained non-significant (*β* = 0.21 per 100 m, 95% CI: −0.03 to 0.45; *p* = 0.089), suggesting results may be influenced by sparse data at the longest distance.

Meta-regression was not performed for the outcome of blood lactate because fewer than 10 studies were available, precluding reliable assessment of effect modification.

#### 3.4.4. Sensitivity analyses

Sensitivity analyses varying the assumed correlation (*r* = 0.50, 0.70, and 0.90) yielded positive pooled effects and did not change the overall conclusion for swimming performance (Supplementary Table S3). For subgroup analyses, sensitivity analyses using alternative assumed within-subject correlations showed that the overall direction of subgroup findings was maintained. In particular, the between-subgroup contrast for caffeine dose remained positive across all assumed values, was borderline at *r* = 0.50 (*p* = 0.052), and remained statistically significant at *r* = 0.70, 0.90, and 0.92 (Supplementary Table S4). The remaining subgroup comparisons were not materially altered and remained non-significant across alternative assumed correlations. Sensitivity analyses using alternative assumed within-subject correlations did not materially alter the interpretation of the swimming performance meta-regression models (Supplementary Table S4). For dose, statistical support remained at most borderline. For ingestion timing and distance, the preferred specification remained linear and no evidence of effect modification was observed across assumptions. Accordingly, these meta-regression findings were interpreted as exploratory.

Besides, the results were also robust across sensitivity analyses varying r from 0.50 to 0.90 in blood lactate, with pooled estimates remaining positive and CIs excluding zero (Supplementary Table S5). In the primary analysis of blood lactate, stroke category and distance category showed evidence of between-subgroup differences. However, these exploratory subgroup findings were more sensitive to the assumed within-subject correlation than the overall pooled lactate effect. Specifically, the between-subgroup differences for stroke and distance were not statistically significant at *r* = 0.50, but became statistically significant from *r* = 0.70 onwards (Supplementary Table S6). The remaining subgroup comparisons were not materially altered and remained non-significant across alternative assumed correlations.

Using a single inverse-variance–weighted effect size per study, the pooled effects were similar to the primary analysis and remained statistically significant for swimming performance (SMD = 0.53, 95% CI: 0.16 to 0.90; Supplementary Figure S3) and blood lactate (MD = 0.78, 95% CI: 0.17 to 1.38; Supplementary Figure S4). Leave-one-out sensitivity analyses suggested that no single study unduly influenced the results for either outcome; pooled estimates remained positive and the overall inference was unchanged across all iterations (Supplementary Figure S5).

In a sensitivity analysis excluding studies using caffeine mouth rinse, coffee, or energy drinks, the pooled results were materially unchanged. For swimming performance, the pooled effect remained significant in the sensitivity analysis (SMD = 0.65, 95% CI: 0.15 to 1.16) and was similar to the main analysis (SMD = 0.57, 95% CI: 0.20 to 0.94). Likewise, for blood lactate, the sensitive analysis yielded a comparable pooled effect (MD = 0.87 mmol/L, 95% CI: 0.13 to 1.61) relative to the main analysis (MD = 0.85 mmol/L, 95% CI: 0.22 to 1.49) (Supplementary Table S7).

## 4. Discussion

This three-level synthesis indicates that acute caffeine ingestion confers an ergogenic benefit for swimming performance (SMD = 0.57; corresponding to an ~1.7% improvement), with a concurrent increase in post-test blood lactate concentration (MD = 0.85 mmol/L). Evidence certainty was low for both outcomes, yet heterogeneity was substantial and mainly between studies, suggesting context-dependent effects rather than uniform benefits across athletes and settings. Exploratory moderator analyses suggested that doses ≥6 mg/kg may be associated with larger performance effects, whereas other subgroup patterns were sparse and should be considered hypothesis-generating for future trials.

### 4.1. Main results

Although several previous reviews have examined the effects of caffeine on swimming performance, their findings have not been fully consistent and their analytical scope has varied substantially. Importantly, while the direction of effect across previous reviews was generally similar, with caffeine tending to improve performance, the statistical support was not equally robust. Among the available meta-analyses, only one review reported a statistically significant overall ergogenic effect [15], whereas several more recent syntheses reported non-significant caffeine-specific estimates, often based on sparse outcome-specific evidence. Our multilevel meta-analysis suggests an ergogenic benefit (pooled SMD = 0.57), consistent in direction with the estimate from the prior review (SMD = 0.20), although the magnitude is larger. Importantly, the present review extends prior work by including additional trials, synthesising a broader range of swimming performance metrics (time- and velocity-based outcomes across different race distances), and applying a multilevel framework to account for multiple non-independent effect sizes within studies. Accordingly, the incremental contribution of the present review lies not merely in updating the pooled estimate, but in providing a broader and more methodologically robust synthesis of the question. When expressed on a ratio scale, the pooled effect corresponds to an average improvement of ~1.71% in performance. Moreover, analysis of data from the Olympic Games indicates that improvements in swimming performance as small as 0.6% are practically relevant as they can influence competition placings [3].

Some of the mechanisms underpinning caffeine’s ergogenic effect include its influence on central nervous system drive, subsequent reduction of perceived exertion and pain, and enhancement of neuromuscular function during high-intensity exercise [38,39]. Caffeine may also facilitate excitation–contraction coupling and increase motor unit recruitment [10,40]. Consistent with an enhanced capacity to sustain higher intensities, our synthesis also showed higher post-test blood lactate concentrations following caffeine ingestion, which may reflect a greater anaerobic glycolytic contribution and/or higher achieved work rates rather than an adverse effect [41]. Although this was a secondary outcome, caffeine increased post-test blood lactate on average (MD = 0.85 mmol/L), with substantial heterogeneity. This is best interpreted as an intensity-related signal, because post-exercise blood lactate is strongly influenced by the achieved workload and is sensitive to sampling timing (with peak values commonly occurring several minutes post-exercise). Although caffeine appeared to improve swimming performance and increase post-exercise blood lactate, the certainty of evidence for both outcomes was low because of inconsistency across studies and limited information size; therefore, these findings should be considered preliminary.

#### 4.2. Moderator analyses

One of the main additional contributions of the present review is the exploratory assessment of effect modification. Previous reviews were generally unable to investigate moderators meaningfully because of limited evidence bases, narrow analytical scope, or the absence of detailed subgroup and meta-regression analyses. Exploratory moderator analyses were undertaken to help explain the substantial between-study heterogeneity and to generate priorities for future confirmatory trials. These analyses should be interpreted cautiously because several moderators are correlated at the study level, some subgroups were sparse, and multiple subgroup analyses increase the likelihood of chance findings. In addition, some exploratory findings were sensitive to the assumed within-subject correlation used to derive paired effect sizes, particularly for blood lactate; therefore, these analyses should be interpreted as signals for future research rather than definitive evidence of moderation. Interpretation was based primarily on formal between-subgroup tests rather than on differences in statistical significance across individual subgroups.

#### 4.2.1. Caffeine dose

With these caveats, caffeine dose appeared to be associated with effect modification for swimming performance. Pooled effects were substantially larger at doses ≥6 mg/kg than at <6 mg/kg. A possible interpretation is that higher doses more reliably achieve concentrations sufficient to elicit central and peripheral actions relevant to performance [42,43], including greater adenosine receptor antagonism, reduced perceived exertion and pain, and enhanced neuromuscular function. Interestingly, this was also the only between-subgroup comparison for swimming performance that remained directionally consistent and statistically supported across most alternative assumed correlations, becoming only borderline (*p* = 0.052) at *r* = 0.50.

In swimming, where the spectrum of submaximal speeds separating key intensity domains is remarkably narrow [44], even small changes in power output and fatigue resistance can translate into meaningful changes in sustainable pace [45]; a dose threshold may therefore translate into more consistently detectable benefit.

At the same time, dose is unlikely to operate in a simple deterministic manner, since inter-individual pharmacokinetics and caffeine sensitivity vary widely [46]. These competing influences provide a biologically coherent reason why subgroup contrasts may be apparent while meta-regression provides only weak support for a smooth dose–response relationship. The dose meta-regression further illustrates the limits of the current evidence base. Although the linear slope suggested a possible positive trend and the comparison of non-linear specifications produced only small differences in model fit with at most borderline likelihood ratio support, these models were constrained by the small number of distinct dose levels and by reliance on between-study variation to identify dose effects. As a result, any apparent curvature or threshold should be treated as exploratory. This caution also applied to the continuous meta-regression models, which were not materially altered by alternative assumed within-subject correlations and continued to provide borderline support (*p* from 0.06 to 0.09) for dose patterning.

By contrast, lactate outcomes did not show clear dose dependence in our moderator analyses, suggesting that post-test lactate changes may primarily reflect the achieved intensity under each protocol rather than a dose-graded metabolic effect.

#### 4.2.2. Distance

Distance showed only borderline evidence of between-subgroup differences in swimming performance, with a tendency toward larger effects in ≥200 m events; however, this pattern was not corroborated by continuous meta-regression, and sensitivity analyses suggested that inference may be influenced by sparse data at the longest distances.

Exploratory subgroup analyses also suggested between-distance differences for lactate outcomes, with larger post-test lactate increases in short distance (<200 m) than middle-long distance (≥200 m) events. This is physiologically plausible because the relative contribution of anaerobic energy provision increases as exercise duration shortens, and high-intensity swimming can elicit substantial post-exercise lactate accumulation; thus, small ergogenic increases in achievable intensity may translate into larger lactate responses in shorter events [47]. This exploratory subgroup signal was more sensitive to the assumed within-subject correlation than the overall pooled lactate effect, as statistical support was attenuated at *r* = 0.50. Accordingly, this finding should be regarded as an exploratory signal rather than definitive evidence that race distance modifies the lactate response to caffeine.

#### 4.2.3. Stroke

Exploratory subgroup analyses suggested larger post-test lactate increases in freestyle than in preferred strokes, whereas stroke did not clearly modify the effect of caffeine on swimming performance. Because freestyle is generally more economical [48], a higher lactate response is unlikely to reflect a stroke-specific increase in metabolic strain at a matched external speed. Instead, it may indicate higher achieved intensity in freestyle trials, where swimmers may attain greater speed and/or stroke rate during self-paced or maximal efforts, potentially facilitated by caffeine’s central and perceptual effects (e.g. reduced perceived exertion/pain and enhanced central drive). Nevertheless, these observations may be confounded by correlated protocol features (distance, trial format, pacing control, and lactate sampling schedules) and should be interpreted as hypothesis-generating, particularly because post-exercise blood lactate typically peaks within minutes and requires repeated sampling to avoid missing the true maximum [49]. This exploratory stroke-related pattern should not be regarded as definitive evidence of stroke-specific moderation, because its statistical support was attenuated under the more conservative assumed correlation of *r* = 0.50.

#### 4.2.4. Other moderators

Other prespecified moderators, including caffeine source, ingestion timing, swimmer gender, and swimmer athlete level, did not clearly modify the performance or lactate effects. However, subgroup and meta-regression estimates were often imprecise due to small numbers within categories and collinearity between protocol characteristics (e.g. formulation co-occurring with ingestion timing). Accordingly, these moderator results are best viewed as inconclusive rather than definitive evidence of no moderation.

### 4.3. Strength and limitations

This review has several strengths. First, we synthesised evidence using a robust three-level random-effects model, which allowed inclusion of multiple outcomes from the same trial while accounting for within-study dependence. Second, we explored clinically relevant moderators (athlete level, caffeine consumption protocol, swimming distance), and we assessed potential small-study effects using both visual inspection of a contour-enhanced funnel plot and Egger’s regression. Third, we included only studies that explored the effect of swimming performance in isolation, not on performance in sports in which swimming is only a single component (i.e. triathlon) [50].

Several limitations should be considered. First, substantial heterogeneity was observed for both swimming performance and post-exercise blood lactate (I²_total_ = 64.7% and 76.5%), indicating meaningful between-study variability; thus, pooled estimates should be interpreted as overall average effects rather than effects that necessarily generalise to all contexts. Accordingly, the present findings should not be interpreted as indicating a uniform ergogenic effect across all swimmers, race distances, or supplementation protocols. Although we examined several moderators, many categories were sparse and the number of distinct values was limited, so subgroup and meta-regression findings should be viewed as exploratory. For blood lactate, the evidence base was smaller (8 studies), and meta-regression was not performed (<10 studies). Second, funnel-plot inspection and Egger’s regression did not indicate small-study effects, but these methods have limited power with few studies and should be interpreted cautiously. Third, risk of bias was generally some concerns, which may contribute to uncertainty. Fourth, although intervention form may complicate mechanistic interpretation, the sensitivity analysis showed that exclusion of caffeine mouth rinse, coffee, and energy drink studies did not materially change the pooled estimates. Accordingly, these intervention forms do not appear to have driven the overall findings in the present review. Finally, results reflect acute caffeine exposure and predominantly male samples; therefore, inferences for female swimmers and chronic supplementation strategies remain limited.

### 4.4. Practical applications

Acute caffeine ingestion appears likely to provide a practically meaningful performance benefit for swimmers under controlled conditions, with the supplementary ratio-of-means analysis indicating an average improvement of approximately 1.7%. However, the substantial between-study heterogeneity suggests that this benefit should not be assumed to occur uniformly across all athletes, events, or testing contexts. Therefore, caffeine use should be individualised and first trialled in training or low-stakes competition before being implemented in an important race.

For additional practical context, we also translated the pooled SMD using the placebo-condition SD from a representative 200 m time-trial study [35], which suggested an approximate improvement of 1.98 s (95% CI: 0.70 to 3.27 s). This estimate was broadly consistent with the 1.7% improvement derived from the lnRoM analysis, which would correspond to an approximately 2.05 s improvement for a 200-m performance of around 120 s. However, this time-based translation should be interpreted cautiously, as it was intended only as an illustrative aid and the overall meta-analysis combined a range of swimming distances and performance metrics.

Among the exploratory moderators, dose provided the clearest indication of potential practical relevance, with larger pooled effects observed at doses ≥6 mg/kg than at lower doses. Even so, the current evidence is not sufficient to define a precise optimal dose, and practitioners should balance the possibility of greater ergogenic effects against the higher risk of side effects and poorer tolerability at larger doses. In practice, swimmers and coaches should prioritise protocol consistency, including dose, timing, and product form, and evaluate the athletes’ response in the specific event and race setting most relevant to competition.

## 5. Conclusion

Acute caffeine ingestion confers a moderate ergogenic benefit for swimming performance under controlled experimental conditions (SMD = 0.57, corresponding to an approximate 1.7% improvement) and is associated with higher post-test blood lactate (MD = 0.85 mmol/L), with low certainty of evidence for both outcomes. Exploratory moderator analyses suggested that doses ≥ 6 mg/kg may be associated with larger performance benefits, whereas other performance moderators were generally inconclusive. For blood lactate, exploratory subgroup patterns suggested larger increases in freestyle and shorter events, but these findings were more sensitive to modelling assumptions and should be interpreted cautiously. Overall, the main pooled effects were robust, whereas moderator findings should be viewed as hypothesis-generating priorities for future research.

## Supplementary Material

Supplementary Material supplementary_fileclean.docx
