# Supplementary material for: Caffeine makes a splash: a systematic review and multilevel meta-analysis exploring the effects of caffeine intake on swimming performance
Source: J Int Soc Sports Nutr. 2026 Jun 21;23(1):2692016. doi: 10.1080/15502783.2026.2692016 (PMC13288720; doi:10.1080/15502783.2026.2692016)
Supplement: Supplementary Material — supplementary_fileclean.docx [file RSSN_A_2692016_SM1602.docx]

**Contents**

[Supplementary file 1 1](#_Toc11419)

[Table S1. 3](#_Toc21075)

[Table S2. 1](#_Toc6083)

[Table S3. 1](#_Toc11614)

[Table S4. 2](#_Toc23992)

[Table S5. 3](#_Toc9118)

[Table S6. 4](#_Toc14125)

[Table S7. 5](#_Toc32415)

[Figure S1. 6](#_Toc8023)

[Figure S2. 7](#_Toc24569)

[Figure S3. 8](#_Toc24641)

[Figure S4. 9](#_Toc19620)

[Figure S5. 10](#_Toc28280)

Supplementary file 1

Methodological rationale for classifying moderators

Athlete level was classified using the Participant Classification Framework (Tier system) and operationalised as trained/developmental (Tier 2) versus highly trained/national-level (Tier 3) based on study descriptions (1).

Stroke was coded based on the stroke tested in each trial and grouped as freestyle versus preferred stroke, as described by the original studies.

Caffeine dose was dichotomised as <6 vs. ≥6 mg/kg as a pragmatic cut-point reflecting commonly used ergogenic dosing ranges. Absolute doses (mg) were converted to mg/kg using the reported mean body mass; accordingly, the two 250-mg trials (2,3) were classified as <6 mg/kg. Mouth-rinse protocols were excluded from the dose categorisation.

Timing of ingestion was grouped as 60 min vs. 30-45 min pre-test, consistent with common supplementation practice and the typical absorption time course (peak concentrations often occurring within ~30–60 min, with inter-individual variability) (4). Effects administered >60 min were not pooled due to insufficient data, and mouth rinse protocols (administered immediately pre-test) were excluded from timing subgroup analyses.

Administration form was coded as reported by the original studies and categorized as (i) capsules/tablets, (ii) drink, or (iii) mouth rinse. Caffeine tablets and capsules were combined into a single administration category because both represent solid oral forms with comparable dosing precision, absorption profiles, and expected ergogenic effects.

Swimming distance was grouped into short (≤100 m) versus middle-to-long distance (>100 m) (5). Subgroup meta-analyses were performed only when at least three effect sizes were available, to avoid unstable pooled estimates being unduly driven by sparse data, consistent with previous reviews (6,7).

Gender was coded according to participant gender reported in each study. Gender-based subgroup analyses were conducted only for effect sizes derived from single-gender (male-only or female-only) samples or from gender-stratified results; mixed-gender samples without gender-specific outcome data were excluded from gender subgroup comparisons.

Blood lactate sampling time (time bin; post-test minutes) was coded for blood lactate outcomes only and grouped as immediate (0 min), 1–2 min, 3–6 min, and ≥10 min post-test. These windows were selected to reflect common post-exercise lactate sampling practice and the expected time course of blood lactate following high-intensity exercise, with early samples capturing initial accumulation, 3–6 min representing a commonly reported window close to peak values in many protocols, and ≥10 min reflecting later recovery/clearance.

Subgroup meta-analyses were conducted only when at least two independent studies were available within a subgroup. All subgroup analyses were conducted on an exploratory basis and should be interpreted as hypothesis-generating rather than confirmatory.

References

1. McKay AKA, Stellingwerff T, Smith ES, Martin DT, Mujika I, Goosey-Tolfrey VL, et al. Defining Training and Performance Caliber: A Participant Classification Framework. Int J Sports Physiol Perform (2022) 17(2):317-31. Epub 20221229. doi: 10.1123/ijspp.2021-0451.

2. Alkatan M. The Effect of Caffeine on Swimming Speed. J Phys Educ Res (2020) 7:24-9.

3. Collomp K, Ahmaidi S, Chatard JC, Audran M, Préfaut C. Benefits of Caffeine Ingestion on Sprint Performance in Trained and Untrained Swimmers. Eur J Appl Physiol Occup Physiol (1992) 64(4):377-80. doi: 10.1007/bf00636227.

4. Guest NS, VanDusseldorp TA, Nelson MT, Grgic J, Schoenfeld BJ, Jenkins NDM, et al. International Society of Sports Nutrition Position Stand: Caffeine and Exercise Performance. J Int Soc Sports Nutr (2021) 18(1):1. Epub 20210102. doi: 10.1186/s12970-020-00383-4.

5. Grgic J. A Meta-Analysis on the Effects of Caffeine Ingestion on Swimming Performance. Nutrition & Food Science (2022) 52(8):1242-53.

6. Clemente FM, Ramirez-Campillo R, Moran J, Zmijewski P, Silva RM, Randers MB. Impact of Lower-Volume Training on Physical Fitness Adaptations in Team Sports Players: A Systematic Review and Meta-Analysis. Sports Med Open (2025) 11(1):3. Epub 20250120. doi: 10.1186/s40798-024-00808-3.

7. Bitsko RH, Holbrook JR, O'Masta B, Maher B, Cerles A, Saadeh K, et al. A Systematic Review and Meta-Analysis of Prenatal, Birth, and Postnatal Factors Associated with Attention-Deficit/Hyperactivity Disorder in Children. Prev Sci (2024) 25(Suppl 2):203-24. Epub 20220318. doi: 10.1007/s11121-022-01359-3

Table S1. Summary of previous meta-analyses examining the effects of caffeine on swimming performance

| Author | Intervention | Study sample | Main findings | Statistical findings |
| --- | --- | --- | --- | --- |
| Grgic. 2022 | Direct comparison  (Caffeine vs Placebo only) | 8 studies  124 swimmers | Caffeine ingestion significantly improved swimming performance.  Cohen’s d: –0.20; 95% CI: –0.32, –0.08; *p* = 0.0008. | ↑ |
| Huang et al.  2025 | Direct comparison  (Caffeine vs Placebo only) | ① 25m swim:  2 studies  13 swimmers  ② 50m swim:  3 studies  41 swimmers  ③ Swim velocity  2 studies  8 and 9 swimmers | No significant difference between caffeine and placebo in the 25 m time.  SMD: -0.14; 95% CI: -0.91, 0.63; *p* = 0.73.  No significant difference between caffeine and placebo in the 50 m time.  SMD: -0.06; 95% CI: -0.50, 0.37; *p* = 0.78.  No significant difference between caffeine and placebo in the swimming velocity.  SMD: 0.47; 95% CI: -1.97, 2.92; *p* = 0.70. | ≈ |
| Huang et al.  2024 | Network comparison  (Different Supplements; isolated caffeine vs placebo) | ① 100m swim:  1 study  8 swimmers  ② 200m swim:  1 study  6 swimmers | No significant difference between caffeine and placebo in the 100 m time.  SMD: -0.07; 95% CI: -1.20, 1.09. Not reported *p* value.  No significant difference between caffeine and placebo in the 200 m time.  SMD: -0.20; 95% CI: -1.06, 0.68. Not reported *p* value. | ≈ |
| Domínguez et al.  2025 | Network comparison  (Different Supplements; isolated caffeine vs placebo) | 3 studies  50 swimmers | No significant difference between caffeine and placebo in the swimming performance.  SMD:  -0.08; 95% CI: −0.47 to 0.32, *p* = 0.70. | ≈ |

Note:↑: Significant difference between caffeine condition and placebo condition (p < 0.05); ≈: No significant difference between caffeine condition and placebo condition (p > 0.05).

[1]Grgic. (2022) A meta-analysis on the effects of caffeine ingestion on swimming performance

[2]Huang et al. (2025) Does caffeine intake enhance physical and physiological performance in swimmers? a systematic review and meta-analysis

[3]Huang et al. (2024) Effects of Different Dietary Supplements on Swimming Performance: A Systematic Review and Network Meta-Analysis

[4]Domínguez et al. (2025) Sport supplementation in competitive swimmers: a systematic review with meta-analysis

Table S2. GRADE Evidence Quality Assessment Table

| CAF  vs  PLA | Study /Patients | Study Design | Certainty of Evidence Assessment | | | | | SMD/MD  [95%CI] | Certainty  of  Evidence |
| --- | --- | --- | --- | --- | --- | --- | --- | --- | --- |
|  |  |  | Risk of Bias | Inconsistency | Indirectness | Imprecision | Other  consideration |  |  |
| **Swimming**  **Performance** | 13/176 | RCT | Not serious | Serious^a^ | Not serious | Serious^b^ | None | **0.57** (0.20 to 0.94) | ⨁⨁◯◯  Low |
| **Blood**  **Lactate** | 8/79 | RCT | Not serious | Serious^a^ | Not serious | Serious^c^ | None | **0.85** (0.22 to 1.49) | ⨁⨁◯◯  Low |
| *GRADE Quality of Evidence and Strength of Recommendations:  High: The panel has high confidence in the estimated effect.  Moderate: The panel has moderate confidence in the estimated effect.  Low: The panel has limited confidence in the estimated effect.  Very low: The panel has very little confidence in the estimated effect.  a. Downgraded one level for inconsistency due to substantial unexplained heterogeneity across studies.  b. Downgraded one level for imprecision because the total sample size was limited and did not meet optimal information size considerations for a continuous outcome.  c. Downgraded one level for imprecision because the total sample size was very small (n = 79) and did not meet optimal information size considerations for a continuous outcome; however, the 95% confidence interval did not cross the null effect, so one level of downgrading was judged more appropriate than two. | | | | | | | | | |

Table S3. Sensitivity analysis for the assumed correlation (r) used to derive change-score SDs for swimming performance. The table reports pooled effect estimates (SMD; Est), standard errors (SE), and 95% confidence intervals from the primary meta-analytic model.

| r | k | Est | SE | CI lb | CI ub |
| --- | --- | --- | --- | --- | --- |
| 0.50 | 28 | 0.277 | 0.085 | 0.089 | 0.466 |
| 0.70 | 28 | 0.332 | 0.101 | 0.111 | 0.554 |
| 0.90 | 28 | 0.517 | 0.154 | 0.180 | 0.854 |

Table S4. Sensitivity of subgroup contrasts and meta-regression findings for swimming performance across alternative assumed within-subject correlations. The table reports between-subgroup contrast estimates (Est), and 95% confidence intervals (CI) , and *p* values from multilevel mixed-effects models fitted under different assumed within-subject correlations.

Panel A. Key between-subgroup contrasts

| **Moderator** | **Contrast** | ***r*** | **Est** | **CI lb** | **CI ub** | ***p*** |
| --- | --- | --- | --- | --- | --- | --- |
| Dose | High vs moderate | 0.5 | 0.321 | -0.003 | 0.645 | 0.052 |
| Dose | High vs moderate | 0.7 | 0.401 | 0.032 | 0.769 | 0.036 |
| Dose | High vs moderate | 0.9 | 0.655 | 0.132 | 1.178 | 0.019 |
| Dose | High vs moderate | 0.92 | 0.725 | 0.156 | 1.295 | 0.017 |

Panel B. Meta-regression sensitivity

| **Moderator** | ***r*** | **Best-fitting form** | ***p* (linear)** | ***p* (quadratic)** | ***p* (spline)** |
| --- | --- | --- | --- | --- | --- |
| Dose | 0.5 | Quadratic | 0.091 | 0.094 | 0.097 |
| Dose | 0.7 | Quadratic | 0.084 | 0.084 | 0.087 |
| Dose | 0.9 | Quadratic | 0.071 | 0.062 | 0.065 |
| Dose | 0.92 | Quadratic | 0.071 | 0.061 | 0.064 |
| Timing | 0.5 | Linear | 0.883 | 0.587 | 0.583 |
| Timing | 0.7 | Linear | 0.884 | 0.606 | 0.602 |
| Timing | 0.9 | Linear | 0.879 | 0.644 | 0.641 |
| Timing | 0.92 | Linear | 0.878 | 0.651 | 0.648 |
| Distance | 0.5 | Linear | 0.812 | 0.495 | 0.534 |
| Distance | 0.7 | Linear | 0.748 | 0.422 | 0.455 |
| Distance | 0.9 | Quadratic | 0.614 | 0.251 | 0.268 |
| Distance | 0.92 | Quadratic | 0.598 | 0.242 | 0.259 |

Table S5. Sensitivity analysis for the assumed within-participant correlation (*r*) used to derive the paired-difference sampling variance for crossover lactate outcomes. The table reports pooled mean differences (MD; Est), standard errors (SE), and 95% confidence intervals (CI) from the multilevel random-effects model.

| *r* | k | Est | SE | 95% CI (Lower) | 95% CI (Upper) |
| --- | --- | --- | --- | --- | --- |
| 0.50 | 24 | 0.863 | 0.296 | 0.162 | 1.563 |
| 0.70 | 24 | 0.864 | 0.283 | 0.195 | 1.532 |
| 0.90 | 24 | 0.851 | 0.269 | 0.216 | 1.486 |

Table S6. Sensitivity of key between-subgroup contrasts for blood lactate across alternative assumed within-subject correlations. The table reports between-subgroup contrast estimates (Est), and 95% confidence intervals (CI) , and p values from multilevel mixed-effects models fitted under different assumed within-subject correlations.

| **Moderator** | **Contrast** | ***r*** | **Est** | 95% CI (Lower) | 95% CI (Upper) | ***p*** |
| --- | --- | --- | --- | --- | --- | --- |
| Stroke | Preferred stroke vs freestyle | 0.5 | -0.154 | -0.37 | 0.061 | 0.151 |
| Stroke | Preferred stroke vs freestyle | 0.7 | -0.192 | -0.372 | -0.013 | 0.037 |
| Stroke | Preferred stroke vs freestyle | 0.89 | -0.331 | -0.455 | -0.206 | <0.001 |
| Stroke | Preferred stroke vs freestyle | 0.9 | -0.349 | -0.468 | -0.229 | <0.001 |
| Distance | Middle vs sprint | 0.5 | -0.145 | -0.36 | 0.07 | 0.175 |
| Distance | Middle vs sprint | 0.7 | -0.182 | -0.361 | -0.003 | 0.046 |
| Distance | Middle vs sprint | 0.89 | -0.323 | -0.448 | -0.199 | <0.001 |
| Distance | Middle vs sprint | 0.9 | -0.342 | -0.462 | -0.222 | <0.001 |

Table S7. Sensitivity analysis excluding studies using caffeine mouth rinse, coffee, or energy drinks. The table reports pooled effect estimates (Est), standard errors (SE), and 95% confidence intervals (CI) from the primary and sensitive meta-analytic models for swimming performance and blood lactate outcomes.

| **Outcome** | **Analysis** | **k** | **Est** | SE | 95% CI (Lower) | 95% CI (Upper) |
| --- | --- | --- | --- | --- | --- | --- |
| Swimming performance | Main | 28 | 0.569 | 0.168 | 0.202 | 0.935 |
| Swimming performance | Restricted | 24 | 0.655 | 0.223 | 0.151 | 1.158 |
| Blood lactate | Main | 24 | 0.852 | 0.269 | 0.216 | 1.488 |
| Blood lactate | Restricted | 23 | 0.869 | 0.302 | 0.130 | 1.607 |


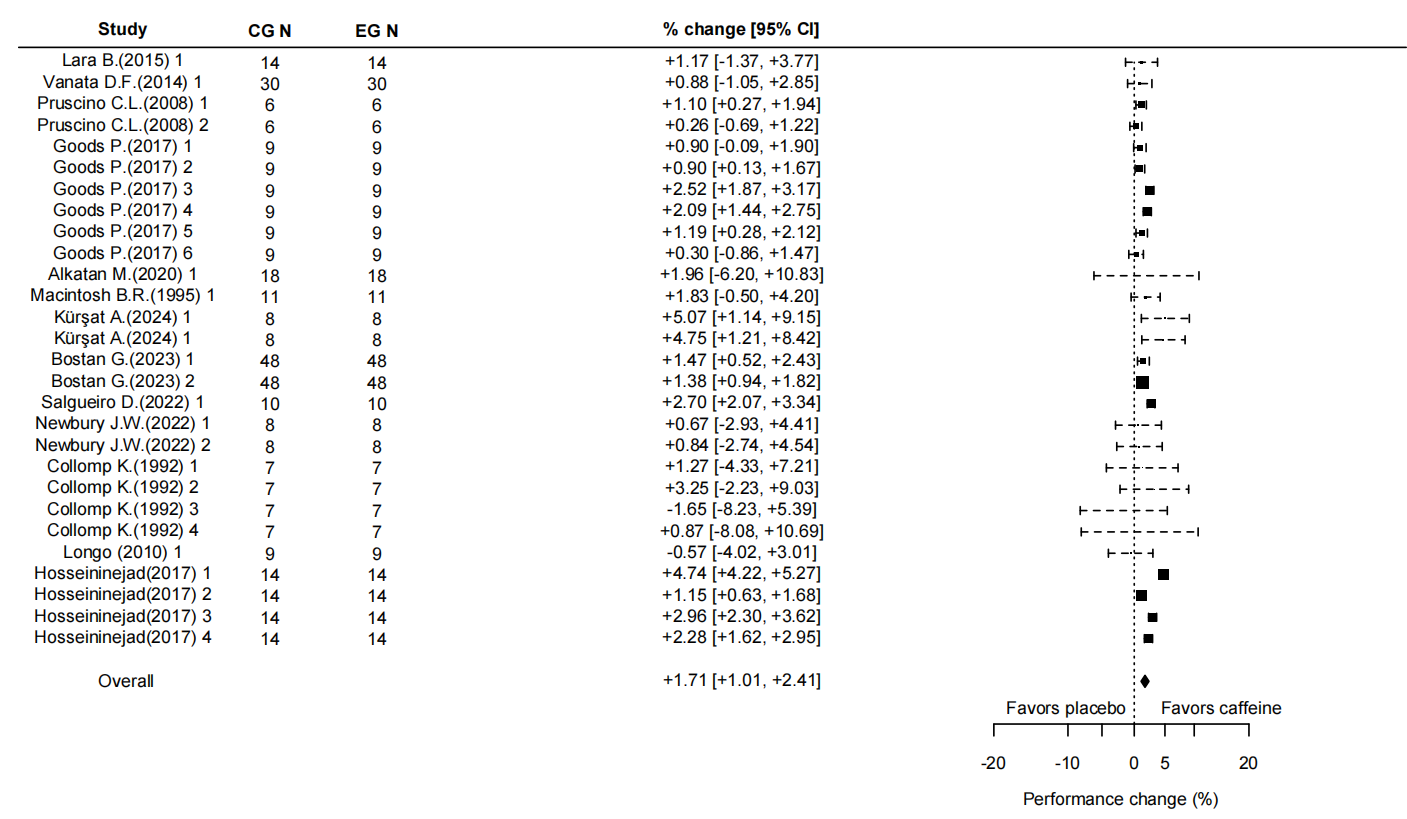


**Figure S1.** Forest plot of the effects of caffeine on overall swimming performance (% change). Squares represent individual effects (size proportional to inverse-variance weight) and horizontal lines indicate 95% confidence intervals. The diamond represents the pooled estimate from the multilevel random-effects model. Positive values indicate improved performance with caffeine. CG and EG denote control and caffeine conditions, respectively; N indicates sample size. Effect directions were aligned so that positive values consistently reflected improved performance.

**
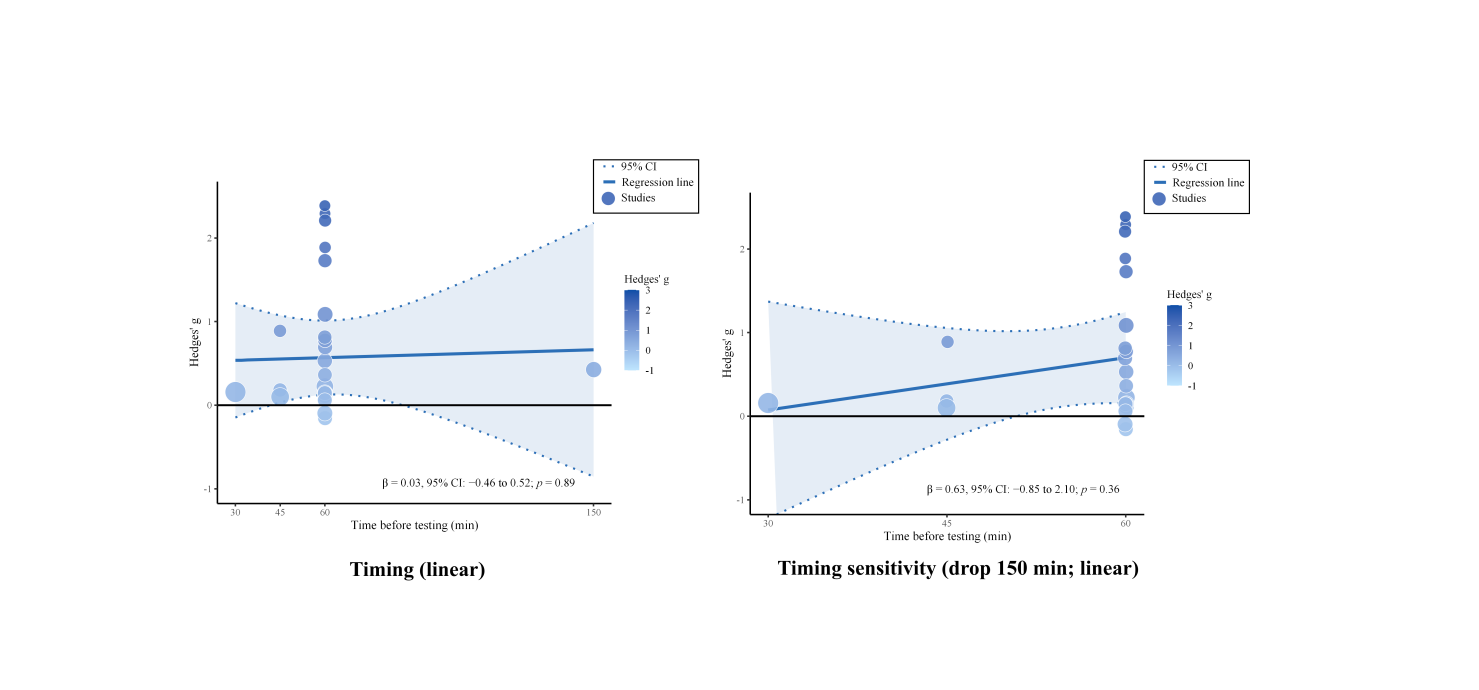
**

**Figure S2.** Meta-regression of ingestion timing (minutes before testing) on the effect of caffeine on swimming performance (Hedges’ g). Circles represent individual effect sizes; circle size reflects inverse-variance weight (precision), and colour indicates the observed Hedges’ g. The solid line shows the fitted linear multilevel meta-regression; the shaded band denotes the 95% confidence interval. The horizontal line marks no effect (g = 0). Left panel shows the quadratic specification fitted to all distances (including 150 min), whereas the right panel shows a sensitivity analysis excluding the single 150-min effect size, after which the results remained unchanged. Model coefficients (β) with 95% CIs and p-values are shown within panels.


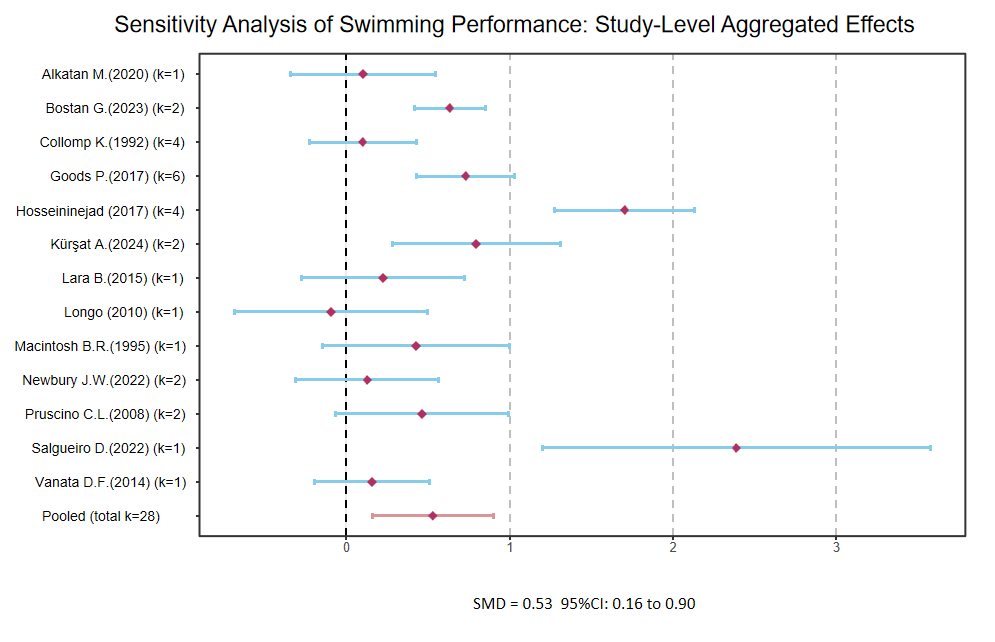


**Figure S3.** Sensitivity analysis using a single inverse-variance–weighted effect size per study for swimming performance. Forest plot showing pooled standardized mean differences (SMD) and 95% confidence intervals derived from a multilevel random-effects model including one aggregated effect size per study.


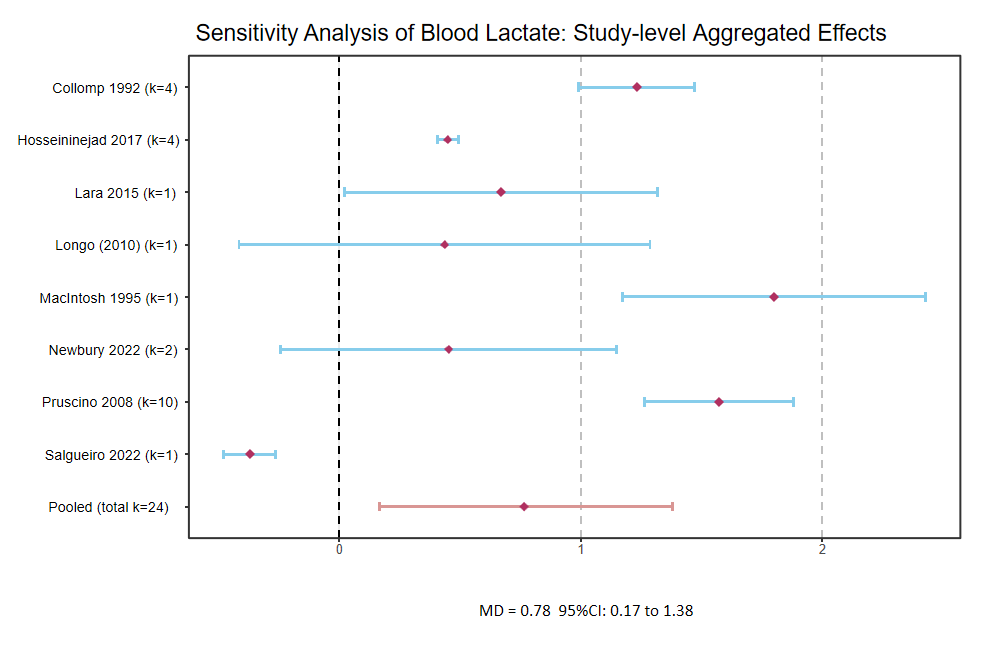


**Figure S4.** Sensitivity analysis using a single inverse-variance–weighted effect size per study for blood lactate concentration. Forest plot showing pooled mean differences (MD) and 95% confidence intervals from a multilevel random-effects model including one aggregated effect size per study.


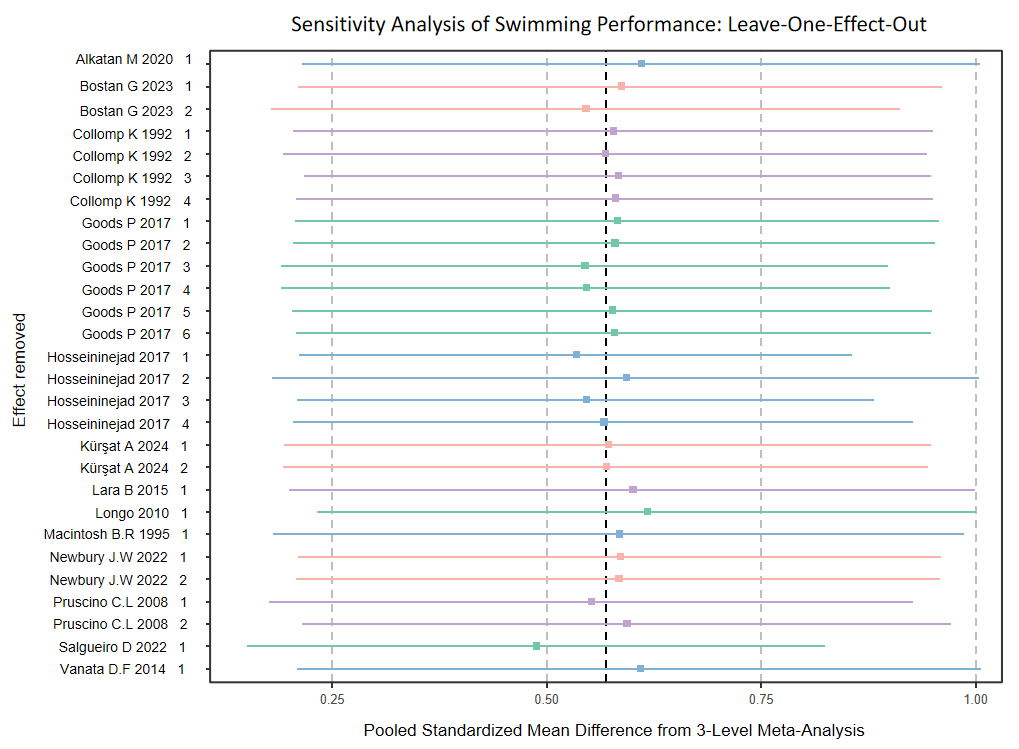


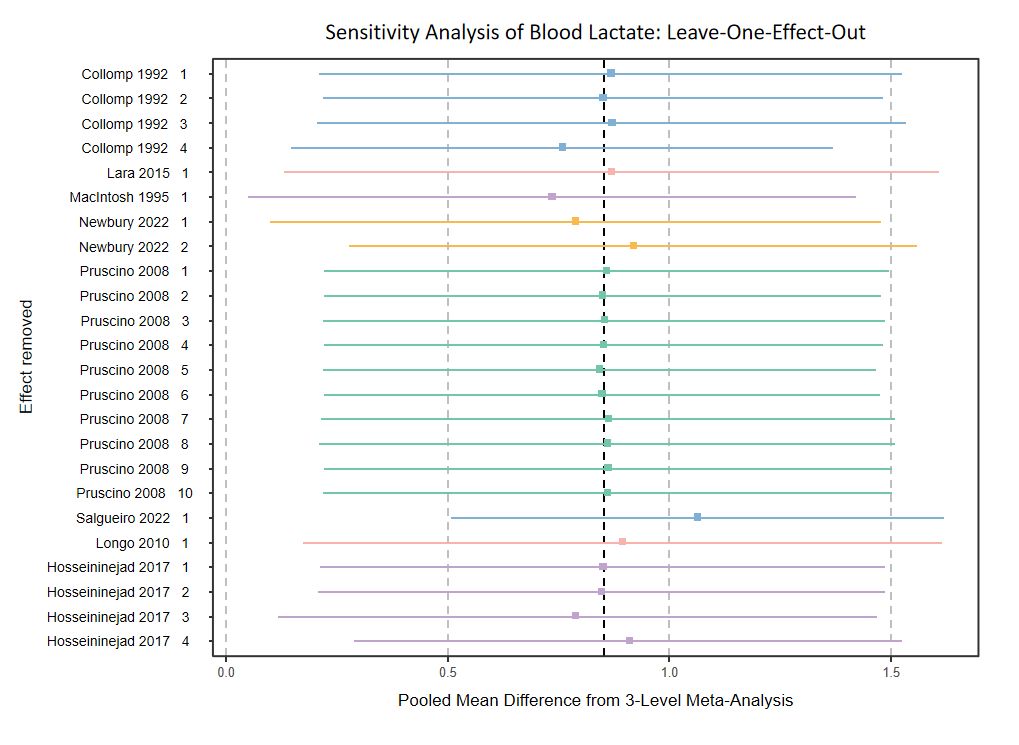


**Figure S5.** Leave-one-out sensitivity analysis for swimming performance and blood lactate outcomes. Each row represents the pooled effect estimate obtained after sequentially removing one study at a time from the multilevel model.
